# Methods to study folding of alpha-helical membrane proteins in lipids

**DOI:** 10.1098/rsob.220054

**Published:** 2022-07-20

**Authors:** Nicola J. Harris, Grant A. Pellowe, Laura R. Blackholly, Samuel Gulaidi-Breen, Heather E. Findlay, Paula J. Booth

**Affiliations:** ^1^ Department of Chemistry, King's College London, Britannia House, 7 Trinity Street, London, SE1 1DB, UK; ^2^ The Francis Crick Institute, 1 Midland Road, London, NW1 1AT, UK

**Keywords:** membrane, protein, lipids, folding

## Abstract

How alpha-helical membrane proteins fold correctly in the highly hydrophobic membrane interior is not well understood. Their folding is known to be highly influenced by the lipids within the surrounding bilayer, but the majority of folding studies have focused on detergent-solubilized protein rather than protein in a lipid environment. There are different ways to study folding in lipid bilayers, and each method has its own advantages and disadvantages. This review will discuss folding methods which can be used to study alpha-helical membrane proteins in bicelles, liposomes, nanodiscs or native membranes. These folding methods include *in vitro* folding methods in liposomes such as denaturant unfolding studies, and single-molecule force spectroscopy studies in bicelles, liposomes and native membranes. This review will also discuss recent advances in co-translational folding studies, which use cell-free expression with liposomes or nanodiscs or are performed *in vivo* with native membranes.

## Graphical abstract

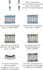

## 1. Introduction

Membrane proteins are ubiquitous macromolecules that reside within dynamic, highly multifaceted cellular membranes. They have two main structural classes, beta-barrel and alpha-helical. This review will focus on alpha-helical membrane proteins, which have hydrophobic alpha-helices that span the lipid bilayer and polar regions which reside outside the membrane. Understanding of the folding of this helical class is advancing and now ranges from *in vitro* studies on artificially denatured proteins and detergent mixtures, through lipid bilayers to co-translational work probing folding events as the protein is made on the ribosome. Diseases caused by misfolded membrane proteins include retinitis pigmentosa, in which misfolding of rhodopsin causes blindness [1], and cystic fibrosis which is associated with mistrafficking and misfolding of the chloride channel CFTR [2,3].

*In vivo* the majority of alpha-helical membrane proteins are integrated into the bilayer co-translationally via SecYEG/Sec61 or via YidC-like insertases [4–7]. The SRP (Signal Recognition Particle) and FtsY mediate the targeting of ribosomes translating integral membrane proteins to the SecYEG translocon for membrane integration [8–10]. Folding of the nascent chain occurs co-translationally, during synthesis by the ribosome and insertion into the membrane, with early folding contacts formed before translation is complete [5,11,12].

**Figure 1.**
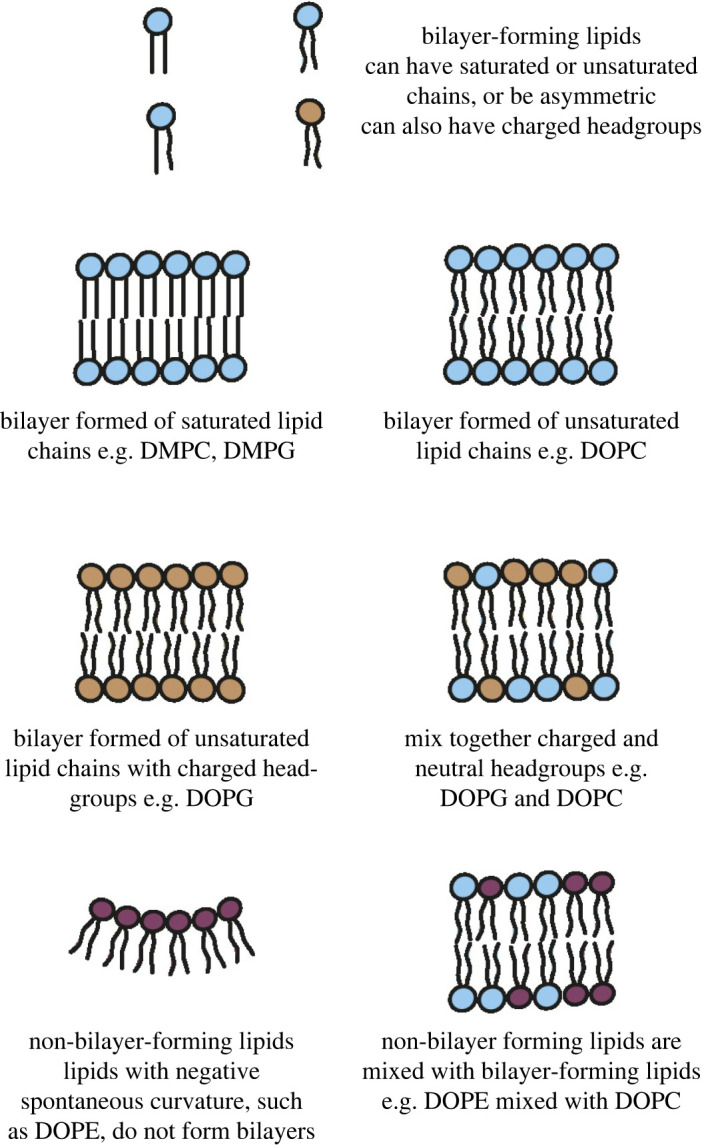
Different lipids form bilayers with different properties. Lipids can be bilayer-forming (e.g. DMPC, DOPC, DOPG) or non-bilayer forming (e.g. DOPE). They can have different headgroups which can be charged or neutral, and can have different chains which can be saturated or unsaturated, or branched. Mixing together different types of lipids produces bilayers with different chemical and physical properties.

*In vitro* studies on alpha-helical membrane proteins began by focusing on protein that had been overexpressed and purified from cells and solubilized in detergents [13–16]. While useful, detergents are unable to replicate the highly complex membrane environment. The lipid composition of the cell membrane is known to affect protein folding [17–22]. Different lipids have different individual properties, such as headgroup charge, chain saturation, chain length and in bilayers give rise to different lateral pressure profiles and phase behaviour [23,24] (figure 1). Most *in vitro* folding studies have used mixtures of synthetic lipids to produce bilayers of varying overall properties, with common lipids including DOPC (1,2-dioleoyl-sn-glycero-3-phosphocholine), a neutral bilayer-forming lipid, DOPG (1,2-dioleoyl-sn-glycero-3-phospho-(1'-rac-glycerol)) which also forms a fluid bilayer but has a negatively charged headgroup, and DOPE (1,2-dioleoyl-sn-glycero-3-phosphoethanolamine). DOPE is a non-bilayer forming lipid, which by itself forms inverted hexagonal phases when dissolved in aqueous solutions, but can be added as a component of a mixed lipid bilayer up to approximately 0.7 mole fraction. This addition increases the stored curvature elastic stress of the membrane, increases the lateral chain pressure and correspondingly decreases the lateral pressure in the headgroup region of the bilayer [25]. DMPC (1,2-dimyristoyl-*sn*-glycero-3-phosphocholine), which is a bilayer forming lipid with shorter saturated chains than DOPC, is also common but forms gel phases at room temperature rather than a fluid bilayer.

**Figure 2.**
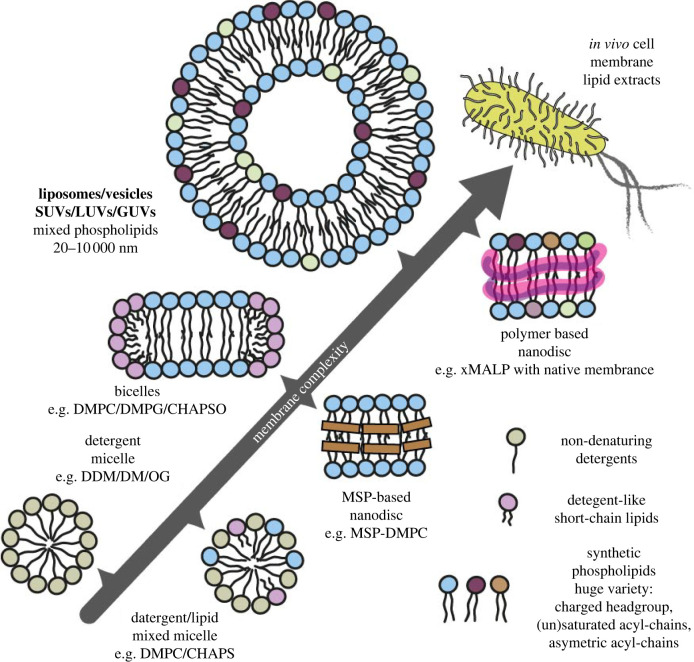
Membrane mimics for membrane protein folding studies. Different membrane mimetics can be used during folding studies. More straightforward systems include detergents or mixed micelles of detergents and lipids. To study folding in lipids, bicelles or membrane scaffold protein (MSP)-based nanodiscs can be used which contain small lipid bilayer sections, with a short-chain detergent around the edges in bicelles, or MSP around the edges in nanodiscs. More advanced folding studies use liposomes, as either small (SUV), large (LUV) or giant (GUV) unilamellar vesicles which contain an inner compartment. This compartmentalization means that liposomes can be used to assess protein topology and for functional assays such as transport assays. The most native lipid system for folding studies is a native lipid extract, or alternatively a novel co-polymer nanodisc can be used which extracts a patch of native lipids from the membrane.

Lipids can have an effect on protein insertion into the membrane [9,18,26–28], the folding rate [27,29,30] and transmembrane (TM) helix topology [18,31–35], and on protein structural stability. Such lipid effects on integral membrane proteins can be linked to protein–lipid interactions, whereby a specific lipid is required for a certain protein conformation or function or can be linked to non-specific interactions that are elicited by the bulk properties of the lipid bilayer described above. Investigating integral membrane protein insertion and folding is affected by the types of synthetic lipid environment used and it is therefore preferential that folding is done in lipid environments rather than detergents. Lipids can be provided in experiments as liposomes, nanodiscs or bicelles (figure 2). The physical properties of the bilayers are slightly different in each of these systems, and different again to the native membrane which has additionally an environment crowded with other proteins. The bilayers in liposomes are relatively unconstrained (very small liposomes will have some imposed curvature stresses) whereas in nanodiscs lipids are encapsulated either by a protein or a polymer ring. Small changes in the absolute value and a broadening of the transition temperature and changes to bilayer rigidity in nanodiscs have been measured compared to the same synthetic lipid mixes in vesicles [36,37]. The size of nanodiscs and bicelles can also vary the bilayer properties. Each has its own advantages and disadvantages, and is amenable to different techniques, which will be discussed further in this review.

There are different ways to approach folding studies in membranes. Classic *in vitro* folding studies use full-length purified protein, so give information on the post-translational folding steps that occur in the lipid bilayer. These *in vitro* studies can investigate TM topology and reconstitution efficiency in different lipids [18], or can unfold and refold a protein by adding and removing a denaturant and measuring the resulting changes in structure [14,17,38–40]. High-detail kinetic and thermodynamic information can be gained from these *in vitro* folding systems [41]. These classic *in vitro* studies lay the groundwork for more advanced folding studies such as single-molecule force-profile studies using either native membranes or protein reconstituted into bilayers composed of synthetic lipids [42–46]. Other progressive methods measure folding during translation by the ribosome, providing information on co-translational insertion into the membrane and subsequent folding [9,26,47]. Co-translational folding studies often use cell-free expression systems with synthetic lipids. All types of folding study have merit depending on what information is needed, and ideally different methods should be applied to each protein to fully understand folding. This review will focus on folding methods which have used bicelles, liposomes, nanodiscs and native membranes, and will first talk about post-translational *in vitro* folding methods before moving onto co-translational folding studies *in vivo*. What is involved in the different methods and what are the advantages and disadvantages of each?

## 2. Protein folding into liposomes

**Figure 3.**
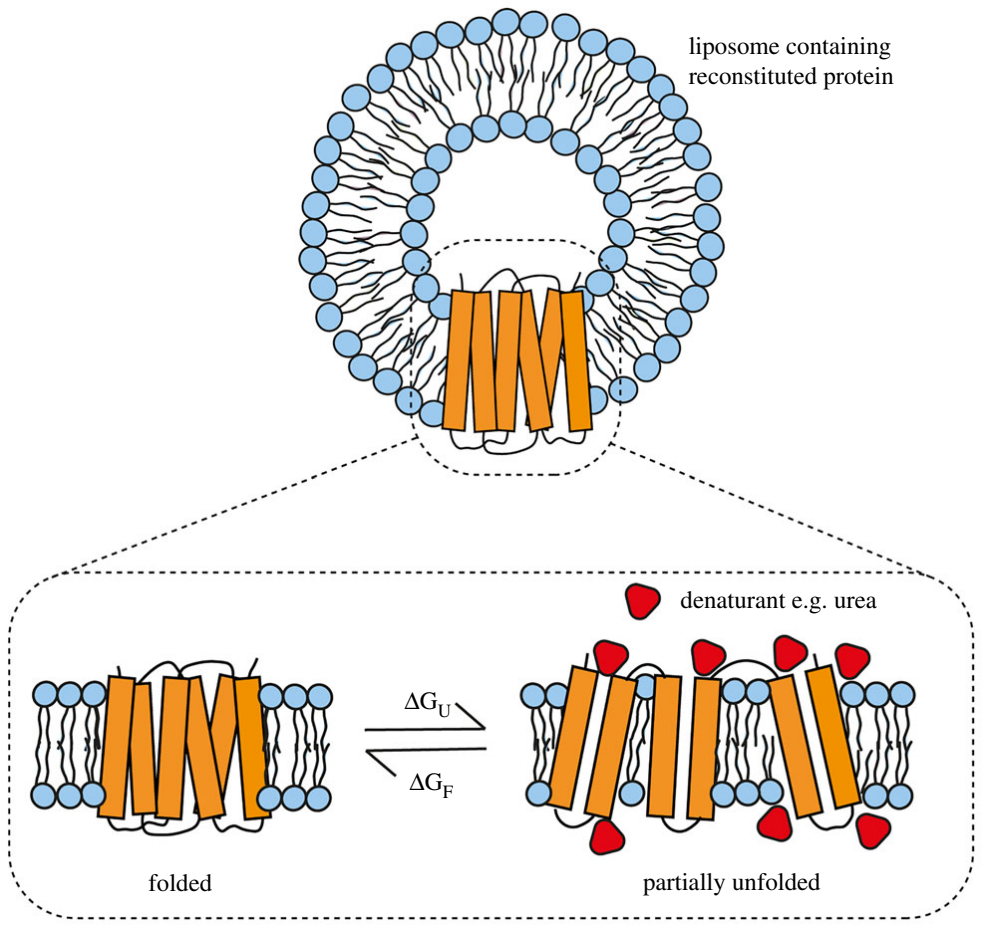
Denaturant folding studies in liposomes. Reversible unfolding of membrane proteins in denaturant can give information on unfolding kinetics and thermodynamics [17]. A fully folded protein is reconstituted into liposomes and a denaturant such as urea is added to partially unfold the protein. Removal of the denaturant can allow the protein to refold. Unfolding and refolding are observed using a variety of techniques, such as by measuring the change in secondary structure by circular dichroism, fluorescence (either intrinsic or via a fluorescent label), or by a protease protection assay. Free energies of unfolding (ΔG_U_) and refolding (ΔG_F_) can be calculated from the equilibrium constant of the fraction of unfolded/folded proteins.

A big advantage of liposomes is that they can easily be made using different lipids, meaning that protein folding into liposomes can be measured in different lipid compositions. There are however technical difficulties that can arise from using liposomes such as scattering artifacts in spectroscopy measurements [48] so they must be used with care. Measuring the reconstitution efficiency, function and stability of the protein in different lipids compositions can give valuable information on the best lipid environment for correct folding. Membrane proteins reconstituted into liposomes can also be unfolded with a denaturant such as urea, and removal of the denaturant can lead to refolding of the protein to its native state (figure 3) [17]. The structure change for each step can then be measured, for example by fluorescence or circular dichroism (CD) spectroscopy. Often this unfolding with denaturant is irreversible, but it is possible to get reversible folding which gives valuable thermodynamic information.

### 2.1. *In vitro* folding in liposomes

It is well established that the Major Facilitator Superfamily (MFS) transporter lactose permease (LacY) topology is dependent on the lipid environment. LacY has two domains and when there are no PE lipids in the bilayer, for example, the N domain of LacY flips in the membrane impairing transport function [35,49]. Much of this LacY topology work was done *in vivo* in *E. coli* genetically modified to have different lipids in their inner membrane [33–35,50]. Recent *in vitro* folding work expanded on this by reconstituting LacY into synthetic liposomes composed of tri-component mixtures of DOPC, DOPE and DOPG, showing that a correct and functional topology of LacY was maintained in DOPC/DOPE mixtures, but the presence of the charged lipid DOPG above a molar fraction of 0.4 resulted in an inverted topology [18].

Proteoliposomes comprised different lipid compositions have been used to investigate whether the reconstitution, folding and function of LacY can be tuned using only the properties of its surrounding bilayer environment [18]. Liposomes made of DOPC, DOPG and DOPE were used to determine the effect of changing global bilayer properties by increasing overall negative surface charge (with a higher fraction of DOPG) and/or increased lateral chain pressure (addition of non-bilayer forming DOPE). Different lipid compositions favoured different properties of the protein, with high lateral chain pressure favouring fast rates of transport and good protein stability but showing poor yield when reconstituting from detergent, and during folding and insertion from denaturant. High surface charge resulted in excellent yields of protein but activity was compromised, only supporting facilitated diffusion down a concentration gradient rather than active uphill transport. This folding work showed that there is a region of lipid composition within this tri-component mix where all of the LacY properties studied are more favourable than not, where the protein is fully functional and with a balance between yield and stability. The related MFS protein GalP showed a similar dependence on DOPE presence as seen for LacY [14]. Excessive DOPE content also hindered the folding yield of GalP, as was observed for LacY. While these experiments show that the conditions can be tuned to make function and folding favourable, a simple tri-component mixture is far removed from the chemical complexity of native *E. coli* membranes [51].

Recent work has shown that alpha-helical membrane proteins reconstituted into liposomes can be unfolded reversibly in denaturant, enabling measurements on the thermodynamics of folding. The bacterial leucine transporter (LeuT) is an SLC6 neurotransmitter transport orthologue that is responsible for the transport of leucine in the hyperthermophilic bacteria *Aquifex aeolicus* [52]. To date, it is the only alpha-helical membrane protein to have its thermodynamic stability measured in a bilayer [17]. The reversible unfolding from the native state of this protein is a fascinating case as this protein also contains multiple molecular knots [17,53,54]. Urea was used as a chemical denaturant to reversibly unfold LeuT both in detergent micelles and in liposomes. By changing the lipid composition of the liposomes, the effects of bilayer properties such as charge and lateral packing pressure on the stability of LeuT could be investigated. Successful refolding was indicated by recovery of at least 95% of the original helical structure, as determined from CD spectroscopy and the recovery of LeuT transport activity. The stability of LeuT could be modulated by the properties of its surrounding bilayer where DOPE or DOPG were found to increase its thermodynamic stability in liposomes [17].

### 2.2. Folding by steric trapping

In addition to reversible denaturant folding for studying the thermodynamic and kinetic properties of membrane protein folding, other methods have been developed using more mechanical forces to study folding. A steric trapping technique can allow the probing of thermodynamic stability, compactness of unfolded state and the unfolding cooperativity of a protein in a near-native environment. Trapping uses a transmembrane protein with added biotin-binding motifs at positions close in space, but distant in linear sequence. On addition of monomeric streptavidin (mSA), which binds biotin, TM helices separate as the protein unfolds. Often, the second mSA is prevented binding by steric overlap and only once the protein unfolds can the second mSA bind [55]. Equilibrium unfolding is therefore controlled by mSA affinity and concentration allowing the protein thermodynamic properties to be assayed across many conditions without the need for additional denaturants. Excess biotin can be introduced to remove the mSA and ensuring reversibility again providing the necessary reaction coordinate for extrapolations of folding energetics. Proteins can also be fluorescently labelled using pyrene-biotin to determine the degree of protein unfolding by observing the increase of fluorescence as pyrene quenching is released as the protein unfolds [55,56].

Bacteriorhodopsin (bR) unfolding using the steric trap was able to accurately probe unfolding in the low SDS concentration region and it was shown that this measured ΔGU is not linear with SDS concentration. bR was therefore shown to have unusually high stability in DMPC/CHAPS bicelles, though lower than previous studies using SDS denaturation [41]. For GlpG a similar trapping method was tested in detergent micelles and the fluorescence change of a pyrene/DABCYL FRET pair during steric trapping were extrapolated and calculated to be 4.7–5.8 kcal mol^−1^, which was significantly lower than the SDS denaturation ΔGU in DDM micelles, which was calculated to be 8.4–8.7 kcal mol^−1^ [57]. In further work, double mSA bound and denatured GlpG was reconstituted from micelles into both bicelles composed of a 3 : 1 mix of DMPC and the negative charged DMPG (1,2-dimyristoyl-sn-glycero-3-phospho-(1′-rac-glycerol)) lipids plus the detergent 3-[(3-cholamidopropyl) dimethylammonio]-1-propanesulfonate (CHAPS), and liposomes of *E. coli* phospholipids [58]. The sterically trapped GlpG remained denatured in both bilayer environments, though could refold to an active conformation on the removal of the mSA. The denatured state in a lipid environment could then be probed using various techniques including proteolysis and double electron-electron resonance spectroscopy. A more or less expanded denatured state was observed depending on the lipid composition.

The CFTR protein TMs 3/4 have been reconstituted into POPC vesicles and have been shown to be amenable to steric trapping methods, this method in lipid bilayers would be a welcome addition to the membrane protein study toolbox [59]. In addition to extending the techniques to bilayers, glycophorin A TM dimerization has also been studied. GpATM reveals dimers that are 4–5 kcal mol^–1^ more stable in 1-palmitoyl-2-oleoyl-sn-glycero-3-phosphocholine (POPC) vesicles than in DM detergent, mutations in dimer interfaces appear to have a greater effect on stability suggesting that the dimers are more organized in a bilayer environment [56].

### 2.3. Advantages and disadvantages

While these *in vitro* measurements are far removed from what happens in the cell, they do allow researchers to attain key thermodynamic parameters. There is also a high degree of flexibility in the types of lipids that can be used, and they can be altered to be optimum for the protein of interest or to mimic the protein's native environment. A notable disadvantage is that these types of folding studies measure an ensemble of states, possibly masking potential important folding intermediates. These types of folding studies do however set a baseline from which other, more complex, studies can be based.

## 3. Single molecule force spectroscopy

**Figure 4.**
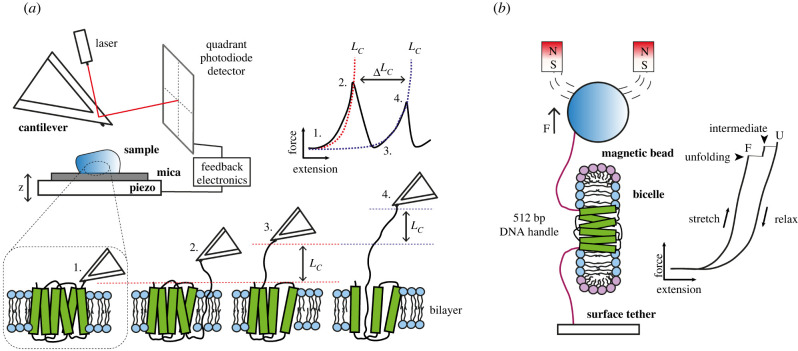
Single-molecule mechanical folding methods. In (*a*), a typical atomic force microscopy (AFM) set-up is shown. A membrane protein sample either in native membranes or reconstituted into liposomes is deposited onto a piezo stage is approached by a sharp cantilever which scans the surface of the sample. The force generated/experienced deflects the cantilever which in turn is detected by a laser and photodiode detector. When the cantilever is retracted from the surface at a constant velocity, a bound membrane protein unfolds in a stepwise manner which is characteristic of the proteins' intrinsic stability. A ‘saw-tooth’ pattern of unfolding is generated where each peak is fit to the worm-like chain (WLC) model of polymer elasticity. The end-to-end length of the unfolded protein or contour length (*L_C_*) is calculated from WLC for each structural segment which unfolds at the given pulling velocity. The *L_C_* can be converted into the number of amino acids unfolded at each event. Alternatively (*b*), a magnetic tweezer set-up can unfold a membrane protein along the plane of the membrane which has been reconstituted into a bicelle or liposome. DNA nano-tethers bind the protein termini to a functionalized magnetic bead and stage. When the pulling velocity is increased, the protein is stretched along the membrane and an unfolding intermediate is observed. This unfolding can be reversible, the protein refolding as the force is decreased.

Single-molecule biophysical techniques allow for the mechanical manipulation of individual membrane proteins [60]. These techniques are capable of resolving the molecular heterogeneity present in a biochemical system, at high temporal and spatial resolutions, and have the significant advantage that they are able to probe folding in membrane environments. Single-molecule mechanical approaches have been employed to probe the stability of individual membrane proteins in bicelles, liposomes, solid supported membranes, and even in native membranes. The main types of instrument used to characterize the mechanical properties of membrane proteins are the atomic force microscope (AFM) and magnetic tweezers (figure 4) [61]. Mechanical folding techniques are collectively known as single-molecule force spectroscopy (SMFS). In these experiments, mechanical forces are used instead of chaotropes as the denaturing agent, and the unfolding pathway of a single protein can be followed using forces applied to it through an AFM cantilever or functionalized bead. The following section will highlight some significant advances made in the mechanical characterization of membrane proteins in lipids.

### 3.1. Mechanical folding with magnetic tweezers

Magnetic tweezers (MT) experiments have been used to investigate the mechanical properties of many globular proteins and have been paramount in probing mechanical unfolding kinetics and elucidating many mechanobiological mechanisms [62–65]. In a typical protein MT SMFS experiment, the molecule of interest is tethered between a superparamagnetic bead and the surface of a glass flow cell (figure 4). Forces are applied to the magnetic bead via a pair of permanent magnets positioned vertically above the flow cell. The excellent force sensitivity and stability of this instrument allows for long term, stable measurements and superb low force resolution (less than 2 pN), provided an appropriate tethering strategy for the molecule of interest is used [62,66]. MT experiments have the advantage that membrane proteins in bicelles or liposomes can be unfolded laterally, with the N- and C- termini separated by the external force (figure 4), disrupting the tertiary contacts between TM helices but not unfolding secondary structure.

There have been recent efforts to employ MT to mechanically unfold two different alpha-helical integral membrane proteins in DMPC/CHAPSO bicelles with DNA nanotethers bound to a magnetic bead and substrate [42,43,60,66]. The ClC chloride transporter of *E. coli* was mechanically unfolded with MT, showing that this protein could be separated into two stable halves that unfolded independently when pulled in DMPC/CHAPSO bicelles [42]. This agreed with the previously suggested hypothesis that the inverted topology of the two domains likely evolved from an ancient genome duplication where the domains folded separately and later fused [42,67,68]. More recently this tweezer system was used to observe the folding the human-β2-adrenergic receptor, which was observed to fold in a strict N-to-C terminal fashion in DMPG/DMPC/CHAPSO bicelles.

The six TM rhomboid protease GlpG has been extensively characterized using a new adaptation of a magnetic tweezer set-up, where the membrane protein was reconstituted into DMPC bicelles and attached to beads using covalently linked DNA handles at the N- and C-terminal. GlpG was shown to unfold in a cooperative manner at high pull forces (25pN), and could be refolded by lowering the force to a few pN [43]. The bilayer environment provided by the bicelle was important for the stability and folding, and switching to detergent micelles or even aqueous buffer alone substantially reduced the unfolding force necessary. Introducing a 0.3 mole fraction of a negatively charged lipid, DMPG, into the lipid fraction of the bicelle did not alter the folding pathway but did substantially increase the probability of refolding. Notably, GlpG has also been unfolded using the same MT technique but in 70 : 30 DMPC : DMPG liposomes. This study found that unfolding in liposomes was similar to that observed in bicelles of the equivalent lipid composition, but refolding was less efficient, dropping to around 15% success [69]. This is the first MT study in a liposome, and the first comparison between different membrane mimetics using MT.

### 3.2. Mechanical unfolding with atomic force microscopy

SMFS investigations of membrane proteins have been dominated by the AFM because of its versatility, allowing researchers to image bilayers, individual proteins, and mechanically unfold individual membrane proteins in both synthetic bilayers and native membranes [70–72]. This means it has more studies in a more native-like bilayers and liposomes than magnetic tweezers, to date.

The basic components of an AFM are shown in figure 4. Proteoliposomes or native membranes are deposited onto a flat mica substrate, generating islands of bilayer which are located by operating the AFM in an imaging mode. The AFM cantilever can be adsorbed to the N or C terminal end of the protein, either by non-specific interaction at contact, or by using protein tags and chemically modified cantilevers. As the cantilever is retracted away from the surface at constant velocity, the peptide backbone experiences a pulling force that increases until it is large enough disrupt tertiary and secondary structure. The corresponding force-distance curve has a characteristic saw-tooth pattern that reveals the mechanical response of that individual protein under an external force. By fitting each of the unfolding steps to the ‘worm like chain’ model it is possible to resolve the number of amino acids released in the unfolding event and assign it to the structure of the protein [73,74]. Multiple mechanical unfolding pathways can coexist, and this single-molecule technique is ideally suited to directly capture and quantify this heterogeneity. AFM SMFS has been used to investigate an array of membrane protein characteristics such as refolding/insertion both in the absence and presence of chaperones [44,45,75], the effects of pH and temperature [76,77], ligand binding [78–80], oligomerization [81], and the effects of surrounding lipid composition on mechanical unfolding and topology [46]. Unfolding and refolding experiments have also been done in native membranes [71], and measurements with folding assisted by the translocon have been made [45,75].

### 3.3. Advantages and disadvantages

AFM has proved to be a versatile tool in characterizing membrane proteins in bilayers. AFM transfers the unfolded segments of the protein into the surrounding aqueous environment, relating it to when helices insert into the membrane during translation [60]. By contrast, mechanical unfolding with molecular tweezers allows for the mechanical distortion of tertiary structure along the plane of the membrane, relating more closely to the stage of folding in which TM helices interact with each other. However, molecular tweezers are currently far behind the versatility of AFM in the study of more physiologically relevant membrane mimetics such as native membranes. Mechanical folding methods offer the advantage that they can get detail on individual molecules, so intermediate folding states can be resolved. They do however require more computational and technical expertise than other folding methods [82–84].

## 4. Methods to investigate co-translational folding

*In vitro* denaturant and mechanical studies have laid the groundwork for advancing to co-translational folding studies. The mechanisms of co-translational folding can be studied either *in vitro* using cell-free expression supplied with a suitable membrane mimetic, or *in vivo*. These folding studies have the advantage that they are more akin to folding in the cell and are denaturant-free, and can be done with synthetic lipids, membrane extracts or *in vivo* with native membranes. Studying co-translational folding helps to elucidate the role that early N-terminal regions of the protein have on the folding of later helices and domains. *In vivo* folding experiments would help us gain a better understanding of how proteins are folded within a cell, and provide an insight into how protein misfolding is implicated in disease [85]. However, increasing the complexity of the environment in which these proteins are studied also increases the general complexity of experimentation. The following sections will describe the current advances in co-translational folding studies of alpha-helical membrane proteins.

### 4.1. Co-translational cell-free methods

**Figure 5.**
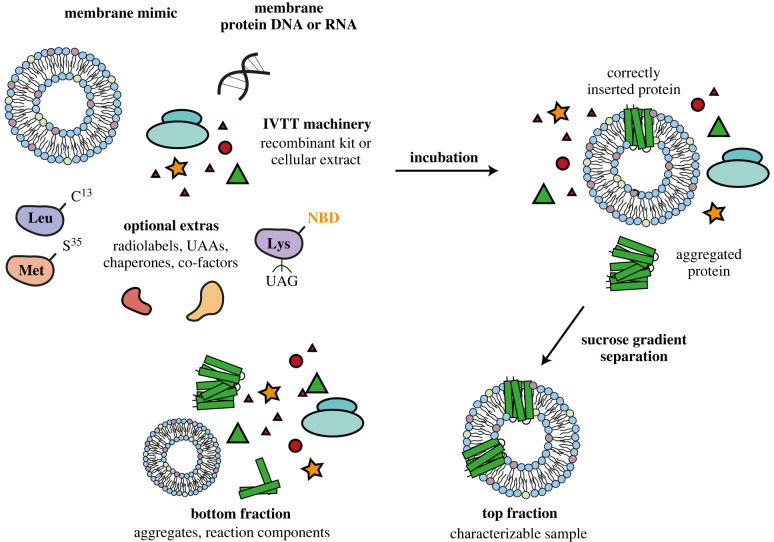
Cell-free expression of membrane proteins. Much of the data on membrane protein co-translational folding come from studies which express the protein cell-free. *In vitro* transcription/translation (IVTT) machinery is produced recombinantly or extracted from a host cell system and mixed with a membrane mimic and the gene for the membrane protein of choice (DNA or RNA). Optional extras to follow the folding of the protein can be added into the reaction mixture, such as radiolabels, fluorescent unnatural amino acids or chaperones. Correctly folded proteins in the membrane mimic can be isolated from the reaction mixture and aggregate/misfolded protein. A sucrose gradient is often used for this when the protein is expressed into liposomes.

Cell-free expression (also known as *in vitro* transcription/translation, IVTT) of membrane proteins has been used for a number of years as a method for the expression of toxic and difficult-to-express proteins [86]. Cell-free expression often uses a cell lysate, which contains the cell transcription/translation machinery and can be derived from *E. coli* (known as an S30 extract), yeast, wheat germ and rabbit reticulocytes [12,87–98]. Alternatively a cell-free system composed of recombinantly expressed and purified transcription/translation components (e.g. PURExpress, [99]) can be used [100–108]. By using cell-free expression, co-translational folding can be followed, and unnatural amino acids or fluorescent probes can be incorporated into the protein as it is synthesized, or the rate of folding can be altered (figure 5).

Most co-translational work to date has addressed insertion of individual TM helices via the translocon, and the topogenesis of these helices has been investigated via a combination of different methods including proteolysis, glycosylation, cysteine accessibility, photocrosslinking and FRET [109–114]. Where co-translational folding studies excel however, is in determining the effect of the lipid bilayer composition on co-translational folding. Different lipids can be supplied during cell-free expression, often as liposomes or nanodiscs. These studies go beyond looking at the insertion of TM helices by looking at how TM helix folding is affected by lipids. A trend is beginning to emerge in which bilayers with headgroup charge (e.g. DOPG) and increased lateral chain pressure (e.g. addition of DOPE) lead to more successful co-translational insertion and folding. These proteins include the *E. coli* rhomboid protease GlpG [9], the β1-adrenergic receptor (β1-AR) [87], the pentameric channel MscL [115,116], and the human GPCR endothelin B (table 1). It is likely that for these proteins the favourable interaction between the TM helices in the nascent chain and the lipid headgroups increases the likelihood that the TMs will insert across a bilayer. Some *in vitro* ensemble folding experiments in liposomes (as summarized above in §2) have also found a preference for DOPG and DOPE, but in these cases, they increase protein stability and are required for correct folding [17,18]. The fact that a correlation has been observed in the lipid preferences obtained with *in vitro* liposome folding and co-translational folding studies suggests that although the methodologies vary in complexity and in their similarities to native conditions, the fundamental results and thus conclusions we draw from these studies may describe the same effects. Each method is equally valuable in furthering our understanding of the fundamentals in protein folding within a lipid environment.

**Table 1.** Summary of optimum lipids for co-translational insertion and folding which have been found from cell-free folding studies.

protein		CF method and membrane mimetic	results from study	ref.
BS-MraY *Bacillus subtilis*	10 TM dimeric enzyme for cell wall synthesis	S30 IVTT with nanodiscs	no preference for **DMPC** or **DMPG**	[112]
DsbB *E. coli*	4 TM disulphide bond reducing enzyme	PURExpress IVTT with liposomes	prefers low lateral chain pressure and neutral headgroups – **DMPC**	[9]
bacteriorhodopsin *Halobacterium salinarum*	7 TM light driven proton pump	S30 IVTT with liposomes	prefers **DOPC** to DMPC, DPPC, DSPC – bilayer thickness and chain saturation important, DOPE inhibits	[110]
Connexin-43 *Rattus norvegicus*	4 TM hexameric small molecule channel	PURE system IVTT and liposomes	prefers **DOPC**, insertion decreases when DPPC or DOPG added	[111]
EC-MraY *E. coli*	10 TM dimeric enzyme for cell wall synthesis	S30 IVTT with nanodiscs	prefers **DMPG** to DMPC, needs 50% PG to function and form dimers	[113]
β1-AR *Meleagris gallopavo (turkey)*	7 TM GPCR	S30 IVTT with nanodiscs	prefers high lateral chain pressure and charge – **PS** or **PG** with **unsaturated *trans* chains**	[80]
endothelin B *Homo sapiens*	7 TM GPCR	S30 IVTT with nanodiscs	prefers high lateral chain pressure and charge – **PS** or **PG** with **unsaturated *trans* chains**	[81]
GlpG *E. coli*	6 TM rhomboid protease	PURExpress IVTT with liposomes	prefers high lateral chain pressure and charge – **DOPG** and **DOPE**	[9]
Opi3 *Saccharomyces cerevisiae*	4 TM^a^ phospholipid methyltransferase	S30 IVTT with nanodiscs	preferred **DOPG/DMPG** to DMPG alone, DMPC not favoured	[114]
MscL *E. coli*	2 TM pentameric mechanosensitive channel	S30 IVTT with liposomes	prefers high lateral chain pressure and charge – **DOPG** and **DOPE**	[108,109]
LacY *E. coli*	12 TM MFS secondary transporter	PURExpress IVTT with liposomes	prefers high lateral chain pressure and charge – **DOPG** and **DOPE**	[26]
XylE *E. coli*	12 TM MFS secondary transporter	PURExpress IVTT with liposomes	**DOPG** highly favoured, **DOPE** also increases insertion yield	[26]

^a^Predicted number of TM segments [115].

While there is an apparent trend emerging for a preference for charged headgroups and lateral chain pressure (table 1), it is not universal across the proteins studied so far. The *E. coli* disulphide bond reducing protein DsbB has been found to insert best into liposomes composed of 100% DMPC, with a reduced insertion yield in the other lipids tested [9]. Similarly, insertion of bacteriorhodopsin (bR) found to be hindered by addition of DOPE to the bilayer. The optimum lipid for insertion was found to be DOPC, when compared to DMPC, DPPC and DSPC, indicating thickness and chain saturation are important properties for insertion of bR [117]. The mammalian channel connexin-43 has also been found to prefer bilayers composed of DOPC, and the amount of protein inserted into bilayers decreased when charged DOPG or the saturated chains lipid DPPC was added to DOPC bilayers [118]. It may be that these proteins insert across the bilayer efficiently enough that increased lateral chain pressure or headgroup charge become inhibitory.

The variation seen in these systems with differing lipid compositions may not be purely down to effects of the membrane protein folding alone but also interactions between the translation machinery and the bilayer. In recombinant systems only non-specific interactions between the ribosomes and the bilayer may contribute, but in more complex extract systems which contain additional soluble chaperones, and in particular with translocon present, further interactions here are likely to be part of the explanation. It is also known that lipids are key modulators of translocase stability and translocation activity. For example, the *E. coli* SecYEG translocon has been shown to require cardiolipin (CL) to function optimally *in vivo* [119]. It has recently been demonstrated that the insertion yield of the bacterial leucine transporter (LeuT) expressed *in vitro* is significantly improved when both SecYEG and CL are present, but not when either CL or the translocon are included alone [120]. This study highlights that particularly when moving towards more ‘native-like’ IVTT systems, the effects of lipid composition on chaperone function, as well as on insertion and folding, must be considered.

Studies using biophysical techniques to study co-translational folding are emerging, often using nanodiscs (figure 2). Nanodiscs are composed of a small planar phospholipid bilayer surrounded by Membrane Scaffold Protein (MSP) [121], and are generally around 10 nm in diameter, depending on the type of MSP used [122]. Nanodiscs have proved a highly useful tool for studies of co-translational folding, as they are amenable to techniques such as mass spectrometry [123,124], and can be immobilized on a surface.

A valuable technique to study the formation of secondary and tertiary structure during co-translational folding is Surface-Enhanced Infrared Spectroscopy (SEIRAS). In SEIRAS, a thin gold layer is deposited onto a silicon prism creating an approximately 10 nm enhancement region in the infrared (IR) signal. Nanodiscs can be tethered to the gold surface, and cell-free expression is initiated on top of the nanodisc layer. The expression, folding and insertion of proteins into the nanodisc bilayer can then be measured using IR spectroscopy [9,47]. SEIRAS has been used to follow the co-translational formation of secondary and tertiary structure of the proteins bR [47], GlpG, and DsbB in DMPC nanodiscs [9]. The folding of all three of these proteins has been characterized with denaturant studies in detergent (GlpG [16], DsbB [125]) and bicelles (bR, [41,126]), and the results of these studies were used to complement and interpret the results from the SEIRAS studies and elucidate the co-translational folding pathway of each protein. SEIRAS represents a powerful new technique for the study of folding as it occurs, and can be used to follow structure formation of different proteins in different bilayers.

### 4.2. *In vivo* folding with force profile measurements

Co-translational folding can also be studied *in vivo*. One method used to probe membrane protein folding *in vivo* is to use translational arrest peptides (APs), in which a stalling peptide sequence is cloned into a region of coding DNA. These stalling sequences are used in nature as a mode of regulation for nascent chain translation [127]. A well-characterized example of a bacterial stalling sequence is SecM [128]. SecM induces stalling in the final codon of its sequence, where a proline alters the ribosomal peptidyl transferase geometry to halt translation until a significant enough force is produced by the nascent chain to release the stall [127,129].

**Figure 6.**
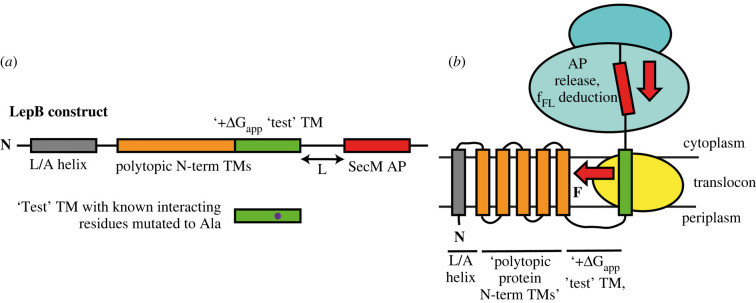
Arrest peptide studies for membrane protein folding. (*a*) The LebB construct used for arrest peptide (AP) force measurements from [125]. A leucine/alanine (L/A) helix was cloned upstream of the polytopic membrane protein to maintain the proteins native N-in orientation. The predicted +ΔG_app_ was calculated for each helix, and a test TM helix was selected from the proteins CaiT, NhaA, EmrD, BtuC and GlpT. Downstream of this at variable length (L) is a SecM AP peptide. (*b*) Schematic of the experimental set-up. The ribosome translates the LepB construct and the helices insert into the membrane via the translocon. Once the AP stalls, the force produced by the partitioning of the test helix into the membrane can release the AP. An f_FL_ value is calculated by the fraction of full-length versus truncated protein produced as determined by western blot.

APs such as SecM are extremely sensitive to tension in the nascent chain and can be applied *in vivo* as force sensors to measure the forces acting upon a nascent polypeptide chain during translation (figure 6) [130–133]. In constructs with highly hydrophobic TM helices, the pulling force of the nascent chain is strong, relieving the AP stall and yielding mostly full-length protein. When the force is low, for example if a TM is not very hydrophobic, the translational arrest will be efficient enough that truncated protein will be made rather than full-length. Placing the AP at different polypeptide lengths can build up a force-profile for a protein by measuring the fraction of truncated protein in relation to fully translated protein by SDS-PAGE at each AP position.

Leader peptidase (Lep) has emerged as an ideal candidate for use in these folding studies. Lep is a two transmembrane *E. coli* membrane protein which has a large C-terminal periplasmic domain that is co-translationally translocated across the inner membrane through the SecYEG translocon. SecM can be introduced near the C-terminal of Lep, and a 19-residue long leucine-alanine based segment varying in hydrophobicity is placed in the C-terminal domain at varying distances upstream of the AP. How the pulling force varies depending on the position of SecM can provide information on the dynamics of transmembrane helix insertion [130]. Measurement of forces which act on charged residues in the nascent chain during translation through the SecYEG translocon have been investigated using this technique using various constructs of Lep [129,130].

The proteins EmrE, GlpG and BtuC have been extensively characterized using the SecM force-pulling assay. A residue-by-residue analysis of each protein combined with mutagenesis and coarse-grained molecular dynamics simulations demonstrated the effect that charged residues, re-entrant helices and surface helices can have on TM integration [132]. These AP assays have also been used to elucidate the contacts that form between TM helices during co-translational folding [131]. These types of experiments have considerably expanded the toolbox for *in vivo* folding studies to measure changes in mechanical forces generated on a nascent chain, in particular during protein folding, membrane insertion and membrane translocation via the translocon [133].

### 4.3. Advantages and disadvantages

Folding studies using cell-free systems are able to look at the effect of the lipid bilayer during co-translational folding, something that cannot be done with folding studies on fully translated proteins. While co-translational studies are limited with regards to which proteins have been studied so far, they still represent a step forward for elucidating folding mechanisms *in vivo* and the information gained can be combined with *in vitro* measurements to get a fuller picture of folding. Structural detail of co-translational folding has been lacking, however recent advances in SEIRAS have started to address this. Most co-translational experiments to date are very limited in what thermodynamic information can be obtained, with only some values for the apparent free energy of insertion provided by the arrest peptide method. In the near future there will hopefully be folding experiments *in vivo* using genetically modified native membranes, and further development for biophysical measurements on *in vivo* samples, to address this gap in thermodynamic information.

## 5. Conclusion and future directions

Folding of alpha-helical membrane proteins has been studied in lipids using multiple methods—*in vitro* folding and denaturant studies, single-molecule force microscopy and cell-free transcription translation. There is no one correct choice for which method to use as it will depend on the information sought. Simplified *in vitro* systems allow for detailed study of thermodynamincs and are more easily adapted. More complex systems are arguably close to the true *in vivo* folding conditions but are not yet advanced enough to measure all desired parameters. These techniques need to improve in a few different directions*. In vitro* denaturant studies need to advance to elucidating the thermodynamics of refolding in lipids, on more proteins from a wider variety of protein classes and organisms. These *in vitro* studies are used as groundwork for co-translational studies, which need improved temporal resolution in order to ascertain folding kinetics and mechanisms. Folding work *in vivo* needs advances in biophysical methods in order to gain more information on how different lipids affect folding pathways. Finally, the vast majority of work to date has been on bacterial proteins and must be expanded to include eukaryotic systems with all the additional complexity that involves.

## Data Availability

This article has no additional data.
